# Research progress in precision medicine for type 2 diabetes based on the GLP-1

**DOI:** 10.3389/fendo.2025.1721842

**Published:** 2026-01-05

**Authors:** Sangui Wang, Chenrong Yuan, Haifeng Wang, Xiyin Ye, Shun Ruan, Li Yi, Wanyong Yang, Zhi Wang, Ni Wang, Jiahao Li, Xiaohui Feng, Yun Li, Yu Tian, Quanlei Wang

**Affiliations:** 1 Department of Surgery, Dongguan Nancheng Hospital, Dongguan, China; 2 Dongguan Institute of Gallbladder Disease Research, Dongguan Nancheng Hospital, Dongguan, China; 3 Department of Internal Medicine, Dongguan Nancheng Hospital, Dongguan, China; 4 Clinical Trial Research Organization, Dongguan Nancheng Hospital, Dongguan, China; 5 Department of Ultrasonography, Dongguan Nancheng Hospital, Dongguan, China

**Keywords:** glucagon-like peptide-1, type 2 diabetes mellitus, l-cell, metabolic surgery, jejunostomy

## Abstract

Diabetes Mellitus (DM) represents a global health crisis, currently affecting approximately 9% of the world’s population. Its prevalence continues to rise steadily, with a noticeable trend toward onset at younger ages. Projections indicate that the prevalence will reach 12% by 2045 equivalent to 820 million cases—positioning DM as one of the most serious public health threats worldwide. Pathogenetically, DM is classified into Type 1 Diabetes Mellitus (T1DM) and Type 2 Diabetes Mellitus (T2DM), with T2DM accounting for over 90% of all cases. T2DM is characterized by pancreatic β-cell dysfunction and insulin resistance, and is recognized as a multisystem metabolic disorder involving pathways such as the gut-brain axis, insulin/peripheral resistance, etc strongly correlated with obesity and cardiovascular diseases. Recent advances in basic medical research and clinical therapeutics have optimized the application of glucagon-like peptide-1 (GLP-1), establishing it as a cornerstone incretin based therapy for T2DM management. In parallel, metabolic surgery has demonstrated significant potential in ameliorating symptoms of T2DM. This article comprehensively reviews current trends in T2DM treatment, the molecular profile of GLP-1, biological characteristics of GLP-1-secreting L-cells, the development of GLP-1-related pharmaceuticals, and advances in metabolic surgery (MS). We searched the primary literature in PubMed, Embase and SciELO from inception to June 2025, using the terms “diabetes”, “type 2 diabetes mellitus”, “glucagon-like peptide-1”, “L-cell”, “metabolic surgery”, “jejunostomy”, “GLP-1 receptor agonists” as well as their combinations. We included basic/mechanistic studies, human observational studies, randomized clinical trials and observational *post hoc* analyses of trials that were relevant to the review topic. The aim is to provide insights and references for future strategies in personalized precision medicine for T2DM.

## T2DM diagnosis and treatment trends

Type 2 diabetes mellitus (T2DM) represents a significant global health challenge, with Asia being a major epicenter of its prevalence (1, 2). China and India have the highest number of diabetes cases worldwide (3). Currently, China reports an incidence rate of approximately 11.7%, affecting around 144 million people. This number is projected to rise to 265 million nationwide by 2045 (2). Compared with other regions, Asian populations experience an earlier onset of T2DM and exhibit a relatively lower body mass index (BMI) (4). Previous studies have established a correlation between increased BMI and higher incidence of T2DM, suggesting that the co-occurrence of obesity and T2DM may become a growing concern in the future (5). The pathogenesis of T2DM is primarily attributed to insulin resistance and pancreatic β-cell dysfunction (6). As the disease progresses, it induces various metabolic and homeostatic imbalances, resulting in persistent dysregulation of glucose and lipid metabolism, compromised vascular integrity and function, and ultimately leading to organ failure and increased mortality. Given the significance of systemic metabolic homeostasis and the intricate pathogenesis of T2DM, addressing these factors is paramount for effective clinical management. The American Diabetes Association now advocates for a complication centric approach to managing diabetes instead of just targeting glucose levels (7). The SELECT and FLOW trials have demonstrated that GLP-1R agonists can significantly reduce the risk of cardiovascular and renal diseases in the studied populations (8, 9). Moreover, the SURPASS trial program has demonstrated that the GLP-1/GIP dual receptor agonist Tirzepatide provides superior glycemic control and weight loss compared to selective GLP-1 receptor agonists like Semaglutide (10, 11). Current management strategies include lifestyle modifications, pharmacological treatments, and metabolic surgery (MS), all aimed at controlling the disease and mitigating its complications. However, sustaining long-term efficacy and ensuring patient adherence remain challenging. With the emergence of GLP-1 related drugs and MS as next-generation treatment options, there is a growing emphasis on developing personalized precision medicine strategies for T2DM patients (12).

## Overview of GLP-1

Glucagon-like peptide-1 (GLP-1) is a polypeptide hormone, which was encoded by the glucagon (GCG) gene on human chromosome 2 and initially consists of 160 amino acids. GLP-1 was first isolated and extracted from human intestinal mucosa in 1985 (13). Following proteolytic cleavage, it yields a biologically active 30-amino acid peptide and is considered a derivative of glucagon (14). GLP-1 mediates its physiological effects by binding to the glucagon-like peptide-1 receptor (GLP-1R). Its key functions include enhancing glucose-dependent insulin secretion, inhibiting glucagon release, and promoting the proliferation and regeneration of pancreatic β-cells. Beyond pancreatic actions, GLP-1 also influences other organs, for example, it delays gastric emptying and suppresses appetite-regulating neurons in the brain, thereby increasing satiety and reducing food intake. Additionally, GLP-1 exhibits potential cardioprotective properties (15). These multifaceted roles make GLP-1 a highly promising therapeutic target for the treatment of type 2 diabetes mellitus (T2DM) and obesity.

## Biology of GLP-1-secreting l cells

GLP-1 is predominantly secreted by L cells, a type of endocrine cell originating from intestinal crypt precursor cells (16). These cells were initially classified based on their morphological characteristics under electron microscopy and their hormone secretion profiles observed via immunofluorescence staining. The intestinal endocrine system comprises not only L cells but also I cells (secreting cholecystokinin, CCK), K cells (producing glucose-dependent insulinotropic peptide, GIP), neurotensin (NT)-secreting cells, and other endocrine cell types resembling pancreatic α-cells (17).These cells exhibit significant morphological and molecular differences. For example, in non-human primate (macaque) colon tissues, L cells display larger and denser nucleoli with a homogeneous appearance, lacking the characteristic halo observed in α-cells. Like most intestinal endocrine cells, L cells are open-type cells with a conical structure, anchored in the basal lamina of the intestinal epithelium. Their microvilli project into the intestinal lumen, while hormone-containing secretory granules are localized on the basal side, facing the capillaries. L cells are distributed along the entire human gastrointestinal tract, with the highest density found in the distal ileum. A lower abundance is present in the colonic mucosa. One study reported that L cell density increases from the proximal duodenum and jejunum toward the jejunum and distal ileum, with higher density in the proximal colon compared to the rectum (18).

L cell distribution may also vary between healthy individuals and those with type 2 diabetes mellitus (T2DM). Patients with severe obesity or T2DM show reduced L cell numbers and altered processing and release of gut hormones (19). Immunostaining reveals more prominent L cell presence in the distal intestinal and colonic regions in T2DM patients. Furthermore, L cells constitute a highly heterogeneous and functionally diverse population (20), with distinct spatial distribution patterns (21). For instance, single-cell transcriptomic analysis of murine intestinal L cells by Leslie L. Glass et al. (2017) uncovered remarkable heterogeneity and functional diversity among these cells (22). In 2019, Helmut Gehart and colleagues used similar approaches to decipher the molecular mechanisms underlying cellular differentiation between L cells and other intestinal endocrine lineages (23). Comparative studies using intestinal tissue staining and single-cell transcriptomics have demonstrated impaired L cell development in colon tissues of diabetic mice, accompanied by reduced GLP-1 secretion and significant downregulation of the transcription factor Foxa1 (24). In 2022, Yan et al. revealed through epigenomic and intestinal organoid models that intestinal Gpr17 deficiency enhances GLP-1 secretion and improves glucose metabolism (25). Most recently in 2024, Joep Beumer and colleagues applied a multi-omics approach integrating genomics, transcriptomics, proteomics, organoid models, and gene editing to delineate the mechanism through which enteroendocrine cells sense ligands and regulate GLP-1 secretion (26).

In summary, from the initial identification of intestinal L cells to contemporary insights into their heterogeneity, plasticity, and regulatory networks, these advances have profoundly enriched our understanding of human physiology and opened new therapeutic avenues for globally prevalent metabolic diseases. With continued progress in multi-omics technologies and reduced operational costs, we anticipate further transformative discoveries that will pave the way for L cell-based precision medicine.

## GLP-1 related drug development process

Naturally secreted GLP-1 peptides are rapidly degraded by the ubiquitous enzyme dipeptidyl peptidase-4 (DPP-4), rendering them unsuitable for direct therapeutic use. GLP-1 exerts its physiological effects by binding to the glucagon-like peptide-1 receptor (GLP-1R) (27). Consequently, both GLP-1 receptor agonists (GLP-1RAs) and DPP-4 inhibitors have emerged as major directions in GLP-1-based drug development for T2DM. Several GLP-1RAs therapies have been approved by the National Medical Products Administration (NMPA), including Exenatide, Liraglutide, Lixisenatide, Albiglutide, Dulaglutide, Semaglutide, and Tirzepatide etc (28). As a result, GLP-1RAs have been incorporated into the 2017 China Guidelines for the Prevention and Treatment of Type 2 Diabetes as a recommended treatment option following metformin. They are also indicated for use in combination therapy. For example, the 2020 China Guidelines recommend that T2DM patients with atherosclerotic cardiovascular disease (ASCVD) or high cardiovascular risk may receive GLP-1RAs in combination with metformin. Advances in understanding the genetic regulatory network of GLP-1 have revealed that the GCG gene, which encodes GLP-1, serves as a core regulator. It interacts with other gastrointestinal peptides, such as glucose-dependent insulinotropic polypeptide (GIP), vasoactive intestinal peptide (VIP), peptide YY (PYY), and the glucagon receptor (GCGR)—to form a functional physiological network. This has spurred the development of multi-agonists, including GLP-1/GIP dual receptor agonists like Tirzepatide. By synergizing GIP with GLP-1 pathways and targeting additional tissues such as adipose, Tirzepatide demonstrates superior glycemic control and weight loss compared to single-target GLP-1RAs (e.g., Semaglutide), setting a new efficacy benchmark (29, 30). The development of triple agonists (e.g., GLP-1/GIP/glucagon agonists like Retatrutide) or other multi-target agents (e.g., GLP-1/amylin/GIP) represents the next frontier in drug discovery (31). Although GLP-1-based therapies can partially mitigate hyperglycemic toxicity in T2DM, they often entail costly lifelong regimens with variable clinical outcomes. There remains a pressing need for more sustainable long-term treatment strategies. Through multi-target activation, innovative delivery methods, and expanded indications, GLP-1-based agents are poised to drive transformative advances in metabolic disease management and chronic care models, demonstrating significant future potential (32).

## Advances in metabolic surgery

Metabolic surgery (MS) includes procedures such as sleeve gastrectomy (SG), Roux-en-Y gastric bypass (RYGB), biliopancreatic diversion (BPD/DS), and jejunoileal bypass (JIB). Initially developed to treat obesity, MS was first proposed by Pories et al. in 1992 as an intervention for type 2 diabetes mellitus (T2DM) (33). Studies have shown that MS in obese T2DM patients often leads to improved glycemic control before substantial weight loss occurs—blood glucose levels frequently normalize within one week post-surgery, while significant weight reduction unfolds over subsequent months. The glucose-lowering effects of MS are attributed to multiple weight-loss-independent mechanisms, including modulation of gut hormones such as glucagon-like peptide-1 (GLP-1).

Patient selection and surgical approach recommendations are based on consensus guidelines from international expert bodies, including the American Society for Metabolic and Bariatric Surgery (ASMBS), the International Federation for the Surgery of Obesity and Metabolic Disorders (IFSO), and the Chinese Society for Metabolic and Bariatric Surgery. Different MS techniques vary in safety, efficacy, and procedural complexity. The choice of procedure should be tailored to individual patient factors, such as BMI, ethnicity, age at diabetes onset, residual β-cell function, and history of prior gastric surgery (**Table 1**). Compared to pharmacological treatments, including GLP-1 receptor agonists metabolic surgeries such as SG and RYGB are associated with higher rates of diabetes remission and more durable glycemic control. Recent evidence indicates that longer preoperative duration of T2DM correlates with reduced likelihood of remission, with each additional year of diabetes leading to a 7% decrease in remission rates (34). Within 1–5 years post-surgery, 30%–60% of patients achieve sustained diabetes remission. Although some patients experience recurrence over the long term, approximately 30% maintain remission, and most exhibit sustained improvements in cardiovascular and metabolic risk factors, diabetic nephropathy, and retinopathy. Many patients experience a prolonged period of improved disease control lasting 5–15 years (35, 36). Despite potential complications such as malnutrition and vitamin deficiencies, ongoing refinements in surgical techniques and perioperative management continue to broaden the applicability of MS for selected T2DM patients. Future research should focus on optimizing patient selection, surgical methodologies, and long-term postoperative care to maximize benefits and minimize risks.

**Table 1:** Comparison of several MS used for T2DM.

Procedure	Laparoscopic jejunoileal lateral anastomosis (LJISSA)	Bileo Pancreatic Diversion (BPD/DS)	Roux-en-Y gastric bypass (RYGB)	Sleeve gastrectomy (SG)
Target population	It is mainly aimed at T2DM patients with normal or high BMI (≤32.5 kg/m²)	It is mainly aimed at patients with extremely high BMI (≥50 kg/m²) and/or T2DM	It is mainly aimed at patients with high BMI (35-50 kg/m²) and BMI ≥30 combined with T2DM	It is mainly aimed at patients with high BMI (35-50 kg/m²) and BMI ≥30 combined with T2DM
Action mechanism	Preserving the continuity of the digestive tract by avoiding tissue removal reduces the risk of severe malnutrition caused by complete emptying. This approach shortens the metabolic pathways for undigested and digested food in the intestines, rapidly stimulating endogenous secretion of GLP-1 from L-cells in the terminal ileum and proximal colon. Postoperative GLP-1 levels can increase by 5-8 times compared to baseline levels.	By removing part of the digestive tract tissue and reducing stomach capacity to restrict food intake, bile and pancreatic juice mix with food only at the terminal ileum, significantly reducing fat absorption (70% reduction in fat absorption, the strongest weight loss effect). Food is exposed to the ileum extremely early, and GLP-1 secretion increases more than tenfold.	The horizontal separation of the stomach body forms a small gastric sac, which limits the intake by reducing the gastric volume. Undigested food enters the distal intestinal tract in advance, stimulating the secretion of GLP-1 and PYY (GLP-1 level can be increased by 5-10 times after surgery), and significantly improving insulin sensitivity.	The removal of part of the digestive tract tissue, the removal of the gastric base area, reduces appetite; food enters the small intestine rapidly, stimulating the secretion of GLP-1 in the distal intestinal tract (about 3-5 times)
Surgical status	Proximal jejunostomy-distal ileostomy	Gastric varix resection; duodenal dissection, anastomosis of the distal small intestine (bile and pancreatic arm) with the terminal ileum;	The body of the stomach was horizontally separated to form a small gastric pouch, which was preserved (about 15-30ml). In addition, the jejunum was dissected and the small gastric pouch was anastomosed with the distal jejunum (Roux's arm); the proximal jejunum (cholangiopancreatic arm) was anastomosed with the Roux's arm at a distance of 75-150cm from the anastomosis.	More than 80% of the gastric wide side was removed, and a banana-shaped gastric tube (capacity 60-120ml) was retained
Availability	The hypoglycemic/weight loss efficacy was weaker than cholecystic resection/duodenal transposition	The effect of weight loss and blood sugar reduction is most significant	Significantly reduce weight, effectively reduce T2DM and related complications, is the most important procedure for obesity and diabetes	Significant weight loss effect; however, the improvement in complications was less significant compared with Roux-en-Y gastric bypass
Safety	Surgical operations, due to intestinal reconstruction, may cause long-term problems with nutrient absorption, but clinical studies are rare and there is no significant data to support it	The surgical operation is relatively complex, with complications including marginal ulceration, anastomotic leakage, intestinal obstruction, and highly complicated lifelong nutritional management; postoperative protein-energy malnutrition (PEM) occurs in up to 25% of cases	The operation is relatively complex, with anastomotic leakage, bleeding and obstruction Venous thromboembolic disease. Internal hernia (most common). Stenosis. Micronutrient deficiency.	The operation is simple and the complication rate is low, but there may be gastroesophageal reflux problem

The jejunoileal bypass (JIB), a classic metabolic surgery (MS) procedure for obesity treatment, gained early prominence due to its non-resective nature. However, it fell out of favor by the mid-1980s owing to severe complications, particularly blind loop syndrome and hepatic failure etc (37). With advances in minimally invasive techniques and procedural refinements such as laparoscopic jejunoileal side-to-side anastomosis (LJISSA), this approach has re-emerged as a potential intervention for type 2 diabetes mellitus (T2DM) (38, 39).

Unlike other MS procedures, LJISSA preserves intestinal continuity and minimizes malabsorption. By creating a side-to-side anastomosis between the jejunum and ileum, it shortens the food transit time, allowing undigested nutrients to rapidly reach the distal ileum. This stimulates L-cells in the ileum and proximal colon to secrete hormones such as GLP-1,GIP etc, enhancing insulin secretion, promoting β-cell proliferation, reducing insulin resistance, improving glucose and lipid metabolism, and potentially inducing satiety signals that contribute to weight loss (40). Studies have shown significant improvements in metabolic parameters—including body mass index (BMI), blood pressure, glycemic levels, insulin resistance, and cholesterol within 3 to 6 months after surgery. Several randomized controlled trials (RCTs) and large observational studies indicate that MS offers superior glycemic control compared to pharmacological and lifestyle interventions in obese T2DM patients. One RCT reported sustained glycemic benefits over 7–12 years of follow-up. A 2016 retrospective analysis of 57 LJISSA cases demonstrated improved glycemic control in all patients, with 34 achieving complete diabetes remission and 23 requiring reduced hypoglycemic medications by 12 months. Patients with a BMI of 28–32 kg/m² experienced weight loss ranging from 7.8% to 20%, though those with a BMI of 24–28 kg/m² showed less pronounced weight reduction (39). In 2021, researchers using T2DM animal models found that following LJISSA surgery, significant changes in the diversity and abundance of gut microbiota regulated amino acid metabolism, thereby improving pancreatic β-cell function. This led to the speculation that modifying the composition of gut microbiota may represent a potential therapeutic strategy for T2DM (41).

A 2025 retrospective study of 78 T2DM patients undergoing jejunoileal bypass showed notable metabolic improvements within 3–6 months post-surgery. Although pancreatic function at 3 months was comparable to baseline, significant enhancement in insulin sensitivity and secretion was observed at 6 months.These benefits were attributed to GLP-1-mediated promotion of β-cell proliferation, inhibition of apoptosis, and improved glycemic regulation (38). The LJISSA technique continues to evolve. A 2025 study introducing a modified anastomosis site reported significantly reduced complication rates, such as diarrhea, small bowel obstruction, and malnutrition from 16.33% to 2.08%, with no compromise in glucose control (42). In summary, LJISSA represents a promising surgical option for T2DM. However, careful patient selection, surgical expertise, and comprehensive postoperative management remain crucial. Further long-term studies are needed to confirm its sustained efficacy and clinical applicability.

## Summary and future directions

The clinical management of type 2 diabetes mellitus (T2DM) continues to pose considerable challenges. Although lifestyle modifications and pharmacological interventions can partially mitigate hyperglycemia and reduce complications, studies show that fewer than 10% of patients achieve complete diabetes remission through behavioral, dietary, and exercise measures alone (43). While conventional drugs such as metformin achieve some degree of glycemic control, their long-term utility is compromised by poor adherence, the need for lifelong administration, and significant side effects. Recent progress in GLP-1 related drugs and MS has markedly advanced T2DM treatment. GLP-1 receptor agonists (GLP-1RAs), designed to mimic native GLP-1 while resisting degradation by dipeptidyl peptidase-4 (DPP-4), have become foundational in T2DM pharmacotherapy. The development of GLP-1-based agents has evolved from single-target drugs to dual- and triple-agonists that also target GIP, amylin and GCGR. By harnessing synergistic mechanisms—including the metabolic flexibility of GIP, the glucagon-suppressing and appetite-suppressing effects of amylin, and the energy expenditure promoted by glucagon—these multi-agonists overcome the efficacy limitations of single-target therapies (44). Future innovations in drug delivery systems and molecular engineering are expected to shift the treatment paradigm from mere glycemic control toward fundamental correction of metabolic dysregulation. amylin.

Harnessing endogenous GLP-1 secretion represents a foundational strategy for achieving long-term remission in T2DM therapy. Endogenous GLP-1 is predominantly secreted by intestinal L cells, which originate from crypt precursors and display spatial heterogeneity along the gastrointestinal tract (18, 45, 46). Notably, studies have identified residual islet cell subpopulations—such as quiescent or dysfunctional β-cells—within the pancreatic microenvironment of T2DM patients. These cells typically do not secrete insulin under physiological conditions but can be reactivated through specific molecular cues to regain differentiation and function (47, 48). GLP-1 has been shown to enhance β-cell redifferentiation and inhibit apoptosis, highlighting its potential as a regenerative therapy (49). The ongoing integration of genomics, transcriptomics, proteomics, metabolomics, and other omics technologies promises to refine GLP-1 based T2DM strategies through detailed characterization of L cell subtypes and gene regulatory networks etc (22, 23).

Metabolic surgeries, including sleeve gastrectomy (SG), Roux-en-Y gastric bypass (RYGB), and laparoscopic jejunoileal side-to-side anastomosis (LJISSA)—leverage the anatomical distribution of L cells to treat T2DM and obesity. These procedures reshape gastrointestinal anatomy to reduce nutrient absorption and stimulate endogenous GLP-1 release, facilitating weight loss and islet cell regeneration. Post-RYGB studies show increased L cell and GLP-1-positive cell density in the ileum and colon, contributing to sustained metabolic improvement via neuroendocrine signaling (43). Variations in intestinal length may enhance physiological and biochemical responses, as stronger hormonal signals from the gut allow nutrient-stimulated gut-derived hormones to reach distal intestinal segments where L cells are located more rapidly (50). At present, there are still disputes regarding the intrinsic cellular and molecular mechanisms of different metabolic surgeries in the treatment of diabetes. The characteristic increase in GLP-1 responsiveness following RYGB may not rely solely on nutrient delivery to distal regions. L cells exhibit stimulus-dependent secretion patterns based on their anatomical location and cellular maturity. For instance, immunohistochemical analyses reveal that while L cells in distal regions (ileum and colon) co-express peptide YY (PYY), those in proximal segments do not produce PYY. Given the existence of multiple L-cell subpopulations, different surgical approaches may influence peptide secretion efficacy by selectively activating specific cell subtypes.

Currently, RYGB and SG are still the mainstream surgical procedures for obesity Despite having a strong effect in treating T2DM. The issues such as visceral resection and surgical complexity in RYGB and SG remain unresolved. The original LJISSA technique was controversial due to complications such as liver injury, diarrhea, and intestinal obstruction associated with anastomotic positioning and intestinal reconstruction. Also in 2025, China’s National Health Commission prohibited the use of “jejunoileal anastomosis” in the treatment of T2DM. Some domestic experts argue that the technique lacks high-quality long-term follow-up data, carries uncertain safety and long-term efficacy profiles, and may lead to severe complications such as liver failure and malnutrition when used in isolation. Future research should prioritize in-depth mechanistic studies on post-LJISSA intestinal physiology and long-term clinical follow-up. Current, Chinese research teams have optimized the LJISSA technique by refining patient selection criteria and the location of the jejunoileal anastomosis. For example, a 2025 study modified the anastomotic site in LJISSA and followed 97 T2DM patients for one year post-surgery. The optimized LJISSA group showed significantly lower rates of complications such as diarrhea, small bowel obstruction, and malnutrition (2.08% vs. 16.33%), with no significant difference in glucose-lowering efficacy compared to the conventional group (42). Despite evidence supporting the efficacy of both bariatric surgery and GLP-1 receptor agonist (GLP-1RAs) in achieving T2DM remission, long-term, head-to-head randomized controlled trials are needed to definitively compare their effectiveness and explore potential synergistic effects of combination therapy (51).

**Figure 1:**
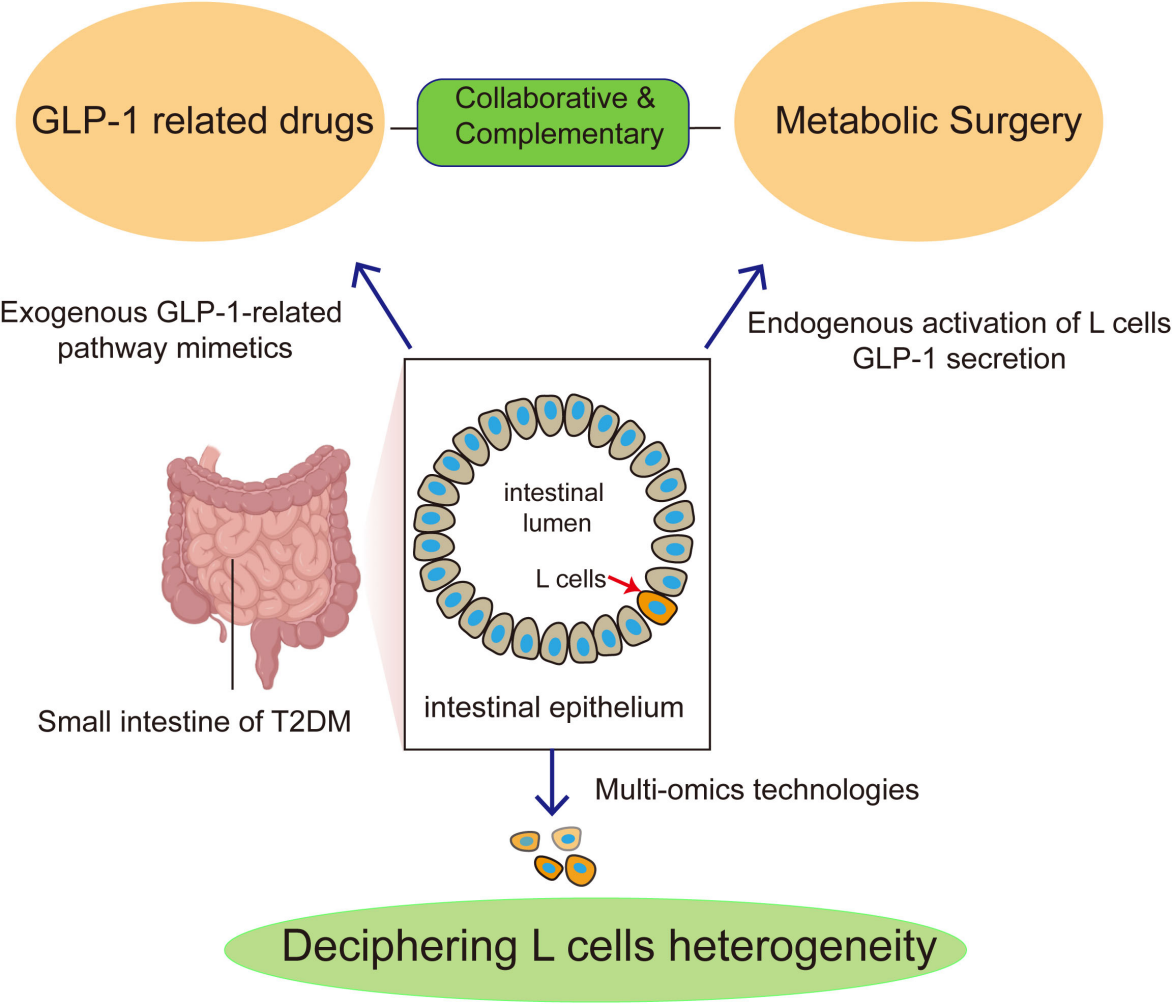
Synergistic approaches to GLP-1 pathway activation in T2DM: From fundamental research to clinical application. Metabolic surgery (MS) is hypothesized to act through endogenous mechanisms, primarily by enhancing GLP-1 secretion from intestinal L-cells. In contrast, GLP-1-related drugs provide exogenous activation of the GLP-1 pathway. MS and GLP-1-related drugs serve complementary rather than substitutive roles. Multi-omics technologies are pivotal for deciphering L-cell heterogeneity, a strategy aimed at elucidating the cellular and molecular mechanisms of the GLP-1 pathway activation and ultimately shaping future research in T2DM.

Treatment concepts for T2DM have evolved significantly both in China and internationally (**Figure 1**). The focus has shifted from solely controlling blood glucose in the early stages to a comprehensive management model that integrates glycemic control, weight management, cardiovascular risk reduction, and improvement of clinical outcomes. GLP-1RAs medications and MS are currently the most promising strategies for treating T2DM. GLP-1RAs medications and MS serve complementary rather than substitutive roles (52, 53). For instance, in specific patient populations, GLP-1RAs can be used as an adjuvant therapy before and after MS to improve glycemic control and manage complications, thereby enhancing surgical outcomes. Conversely, the implementation of MS can reduce the required dosage of GLP-1RAs (54). A recent systematic review has demonstrated that MS consistently yields superior outcomes compared to pharmacologic interventions (such as GLP-1RAs) and intensive lifestyle modification for glycemic control, weight loss, and metabolic improvements in patients with T2DM (55). Previous meta-analysis study suggested that MS provides superior cardiovascular protection and survival outcomes compared to GLP-1RAs in obese and T2DM patients (56). Although MS demonstrates greater efficacy, it still faces significant scalability limitations. In contrast, GLP-1RAs and GLP-1 based multi-agonists offer a safe and widely used therapeutic alternative that can reach a far greater number of T2DM patients (57). Advances in clinical diagnosis and treatment of T2DM rely on breakthroughs in fundamental research. Thus, future studies leveraging animal models, multi-omics technologies to elucidate cellular and molecular network mechanisms of GLP-1 based T2DM therapies will undoubtedly advance personalized precision medicine for T2DM.
